# Healthy lifestyle behaviors and risk of cardiovascular diseases among nursing faculty during COVID-19 Pandemic

**DOI:** 10.1590/0034-7167-2022-0372

**Published:** 2023-10-09

**Authors:** Ayad Majid Mousa Al-Mayahi, Mohammed Baqer Al-Jubouri, Sabah Abdullah Jaafar

**Affiliations:** I University of Baghdad, College of Nursing. Baghdad, Iraq; II University of Al-Muthanna, College of Nursing. Al-Samawa, Iraq

**Keywords:** Pandemics, Disease, Healthy Lifestyle, COVID-19 Pandemic, Cardiovascular Risk, Pandemia, Estilo de Vida Saudável, Comportamento, Doenças Cardiovasculares, COVID-19, Pandemia, Estilo de Vida Saludable, Conducta, Enfermedades Cardiovasculares, COVID-19

## Abstract

**Objective:**

Cardiovascular diseases are the first ranked cause of death worldwide. Adhering to health promoting lifestyle behaviors will maintain an individual’s cardiovascular health and decrease the risk of cardiovascular diseases.

**Methods:**

In this descriptive study, 150 nursing faculty were surveyed via a non-probability (purposive) sampling method to assess their adherence to health promoting lifestyle in order to know the risk of cardiovascular diseases. The Arabic version of Health-Promoting Lifestyle Profile II (HPLP-II) was used to achieve this goal.

**Results:**

Seventy-two nursing faculty completed the survey. The results indicated that the study sample had moderate level of health promotion based on Health-Promoting Lifestyle Profile II.

**Conclusion:**

Nursing faculty are at risk of developing cardiovascular diseases based on their health promoting lifestyle behaviors as they scored low level of *“health responsibility*”, *“physical activity”*, and *“stress management* “. Encouraging healthy behaviors is recommended to prevent chronic diseases such as cardiovascular diseases.

## RESUMO

**Objetivo:**

Doenças cardiovasculares são a principal causa de morte no mundo. A adesão a comportamentos que promovam a saúde contribuirá para manter a saúde cardiovascular de um indivíduo e diminuir o risco de doenças cardiovasculares.

**Método:**

Neste estudo descritivo, 150 professores de enfermagem foram investigados através de um método de amostragem não probabilístico (intencional) para avaliar sua adesão a um estilo de vida que promova a saúde, a fim de identificar o risco de doenças cardiovasculares. A versão árabe do instrumento Health-Promoting Lifestyle Profile II (HPLP-II) foi utilizada para atingir este objetivo.

**Resultados:**

Setenta e dois professores de enfermagem preencheram a pesquisa. Os resultados indicaram que a amostra do estudo tinha um nível moderado de promoção da saúde, com base no instrumento Health-Promoting Lifestyle Profile II.

**Conclusão:**

Os professores de enfermagem correm o risco de desenvolver doenças cardiovasculares com base em seus comportamentos de promoção da saúde, uma vez que eles obtiveram baixo nível de responsabilidade com a saúde, atividade física e gerenciamento do estresse. O incentivo de comportamentos saudáveis é recomendado para prevenir doenças crônicas, tais como as doenças cardiovasculares.

## RESUMEN

**Objetivo:**

Las enfermedades cardiovasculares son la primera causa de muerte en todo el mundo. La adopción de hábitos de vida que promuevan la salud mantendrá la salud cardiovascular del individuo y reducirá el riesgo de enfermedades cardiovasculares.

**Métodos:**

En este estudio descriptivo, se encuestó a 150 profesores de enfermería mediante un método de muestreo no probabilístico (intencional) para evaluar su adhesión a un estilo de vida promotor de la salud con el fin de conocer el riesgo de enfermedades cardiovasculares. Para ello, se utilizó la versión árabe del Health-Promoting Lifestyle Profile II (HPLP-II).

**Resultados:**

Setenta y dos profesores de enfermería completaron la encuesta. Los resultados indicaron que la muestra del estudio tenía un nivel moderado de promoción de la salud basado en el Health-Promoting Lifestyle Profile II.

**Conclusión:**

Los profesores de enfermería corren el riesgo de desarrollar enfermedades cardiovasculares en función de sus comportamientos de promoción de la salud, ya que obtuvieron una puntuación baja en responsabilidad de salud, actividad física y gestión del estrés. Se recomienda impulsar comportamientos saludables para prevenir enfermedades crónicas como las cardiovasculares.

## INTRODUCTION

Health is a process which can be changed sophistically and dynamically. Indeed, health can be affected by person’s lifestyle. As a result; stated that to maintain health, individuals should practice health promoting lifestyle behaviors^(1)^. Health promoting lifestyle behaviors (HPLBs) are activities that include health responsibilities, physical activities, nutrition, spiritual growth, interpersonal relationships, and stress management^(2-3)^. Indeed; by adhering to HPLB, an individual can attain healthy life, and the result is decreasing the risk of diseases^(3)^.

World Health Organization [WHO], (2021a) mentioned that physical activity is healthy measure that has controlling effect in non-communicable diseases (NCDs) such as cardiovascular diseases (CVDs), diabetes mellitus, or hypertension. In addition, it can be used as a tool for prevention and management of NCDs physical inactivity has multiple negative aspects, the most important aspect is population health and quality of life. Furthermore, physical inactivity is a risk factor for CVDs, and it can raise the risk of CVDs by 20-30%^(4)^.

CVDs are the first ranked cause of death worldwide^(5)^. CVDs encompass wide range of diseases including heart and blood vessels which are, however not limited to, coronary heart disease (CHD), rheumatic heart disease, and, cerebrovascular disease^(5)^. Lv et al. (2017) stated that practicing physical exercise, eating healthy diet, maintaining normal body weight, and quitting smoking were associated with a decrease in CVDs and ischemic stroke^(6)^. Indeed, poor healthy lifestyle behaviors lead to poor cardiovascular health^(7)^. Furthermore, Eshah (2011) mentioned that adherence to healthy lifestyle plays a significant role decreasing the mortality and morbidity rate of CHD^(2)^.

Hu et al., (2020) have found that individuals’ lifestyle has been affected negatively in last year because of the pandemic of Coronavirus disease 19 (COVID-19)^(8)^. WHO (2021 b) stated COVID-19 as a communicable disease which is caused by a newly generation of coronavirus. The COVID-19 virus spreads primarily through droplets from infected person by coughing or sneezing. WHO recommended social distancing and avoiding crowds for the most countries worldwide to decrease spread of infection with the virus. These restrictions obviously can affect people’s lifestyle negatively^(9)^. Although, WHO (2021 b) recommended practicing HPLBS during the pandemic of COVID-19 by exercising, eating healthy diet, and avoiding fear and stress to maintain individual’s health^(9)^. Individuals during the pandemic of COVID-19 had low adherence to physical activity and regular sleep at night Thus, the pandemic of COVID-19 may negatively affect individuals’ healthy lifestyle, and this may increase the risk of CVDs in individuals. Individuals are different in each society, and faculty members are a part of each society. During the pandemic, nursing faculty are doing their best by keeping the educational process alive. In fact, they are preparing the front-line soldiers for future. Indeed, their lifestyle may be affected as they use different ways to teach and maybe different ways to live. COVID-19 is a new pandemic that can affect many body organs in different aspects (physical, psychological, or social)^(10)^. On the other hand, individuals’ lifestyles can be changed during this pandemic, and this may affect their health^(11-12)^. Indeed, not all individuals in the community have the same knowledge about health, diseases, and risk factors. Nursing faculty are a part of the community who are aware of diseases and their risk factors because of their profession. However, some unpredicted factors (such as COVID-19 pandemic) may affect their health indirectly by affecting their lifestyle. For this reason, assessing nursing faculty’s lifestyle behaviors can be crucial to prevent diseases (especially cardiovascular diseases). For the mentioned reasons, the researchers in this study aimed to assess the adherence to health promoting lifestyle in terms of knowing the risk of CVDs among nursing faculty during the pandemic of COVID-19^(7,13)^.

The conceptual framework for this study was guided by Pender’s health promotion model who declared that individuals can attain optimal health and wellbeing through practicing health promoting behaviors in their lifestyle such as healthy diet, regular exercise, and adequate rest^(3)^. Also, the researchers hypothesized that nursing faculty do not adhere to HPLBs and they are at risk of CVDs during the pandemic of COVID-19.

## OBJECTIVE

Assessing the adherence to health promoting lifestyle in terms of knowing the risk of CVDs among nursing faculty during the pandemic of COVID-19.

## METHODS

### Design

This descriptive study was conducted to assess the adherence of nursing faculty to health promoting lifestyle in order to know the risk of CVDs among nursing faculty during the pandemic of COVID-19. HPLBs are the independent variables in this study and the dependent variable is risk of CVDs.

### Setting and Data Collection

One hundred ten Iraqi nursing faculty in eight different universities (University of Kirkuk, University of Mosul, University of Baghdad, University of Kufa, University of Al-Muthanna, University of Misan, University of Dhighar, and University of Basra) distributed in eight different provinces from north to south of Iraq (Mosul, Kirkuk, Baghdad, Najaf, Al-Muthanna, Misan, Dhighar, and Basra) who met the inclusion criteria were asked to be participates in this study. The study’s purpose was explained to the participants. Also, an informed consent was given to the participants who agreed to participate in this study. All these processes were done online via Google Form. If a participant would not wish to sign the informed consent, filling the questionnaire is considered as an agreement, and this was explained in the online form. The anonymity was ensured in the online form. Answering to the all items in the questionnaire took about 9 to 18 minutes. The participants were noted that they can withdraw from the study at any time they wish. Also, there would not be any consequences if they do not submit the form. From the total 110 distributed surveys, 72 participants returned it completely with a response rate of 65.4%.

### Ethical consideration

To ensure the ethical consideration, the protocol of this study was submitted to Institutional Review Boards (IRBs) at eight universities (University of Kirkuk, University of Mosul, University of Baghdad, University of Kufa, University of Al-Muthanna, University of Misan, University of Dhighar, and University of Basra). The IRB was obtained from these universities to collect the data. The approval protocol’s number is 644 on March 18, 2021. On the other hand, participants’ privacy was protected by providing confidentiality and anonymity.

### Participants

Iraqi nursing faculty who teaches at Iraqi universities were the population in this study. Those who met the inclusion criteria were considered as the target population in this study. The inclusion criteria were nursing faculty who earned Master or doctorate degree. Also, nursing faculty who teach in private universities were excluded as the study sample includes nursing faculty from governmental universities only.

### Design and Sample

In this study, 110 nursing faculty were surveyed via a non-probability (purposive) sampling method. This method is considered as a suitable sampling method to select the sample from the target population because not all Iraqi nursing faculty have an equal chance to be surveyed in this study. The minimum sample size was 110 as calculated based on the population of nursing faculty in Iraq (330) and the confidence level of 80% with 5% margin of error. The collected sample was 72 in this study with a response rate of 65.4%. The low response rate can be due to busy schedule of nursing faculty.

### Instrument

The questionnaire consists of demographics and the Arabic version of Health-Promoting Lifestyle Profile II (HPLP-II). The demographics consists of age, gender, marital status, monthly income, academic degree, scientific position, affiliation, years of experience, smoking, drinking alcohol, chronic diseases, and Body Mass Index (BMI). The BMI is calculated based on participants’ weight and height, and it is classified in four groups; underweight, normal weight, overweight, and obese with BMI of (below 18.5, 18.5-24.9, 25-29.9, above 30), respectively^(14)^.

HPLP-II is a tool that measures health-promoting lifestyle which developed by Walker, Sechrist, and Pender (1987)^(15)^. HPLP-II contains six domains; *“health responsibility*” (9 items), *“physical activity”* (8 items), “*nutrition*” (9 items), *“spiritual growth*” (9 items), *“interpersonal relations*” (9 items), and *“stress management”* (8 items) with a total of overall 52 items. The mean score for each domain is calculated by the summation of all items in that domain divided by the items’ number. The overall mean score of HPLP-II is obtained by the summation of all items divided by the all items’ number. The psychometric properties of the Arabic version of HPLP-II were tested by Eshah (2011)^(2)^. The results showed that this version is a valid and reliable tool^(2)^. The permission to use the Arabic version was obtain via email from the copyright owner. The instrument is in Arabic, and Iraq is an Arab country with Arabic culture. For this reason, the instrument is appropriate to be used in Iraq. Based on Eshah (2011), the higher score indicated the higher level of health promotion. The mean score of each domain is calculated as low (less than 2.5) moderate (2.5 - 3), or high (more than 3)^(2)^.

### Data Analysis

To accomplish the data analysis, the Statistical Package for the Social Sciences (SPSS) 21 software was used. Participants’ demographics and health status variables were analyzed by descriptive statistics in terms of frequency, mean and standard deviation. To test difference between HPLP-II domains with Participants’ demographic and health status variables, the following parametric statistical tests were used based on level of measurement which are independent two-sample t test for dichotomous independent variables, and analysis of variance (ANOVA) test for polychotomous independent variables.

## RESULTS

Seventy-two nursing faculty completed the survey and entered the phase of data analysis. The mean of study sample’s age was 37.84 years with a Standard Deviation (SD) of 6.95 years. Almost half of the participants were male (51.4%) and a little less were female (48.6%). The majority of the study sample were married (83.3%), and 65.3% of them mentioned that their monthly income is enough. Regarding participants’ academic degree, exactly half of them (50%) hold a master’s degree and the other half (50%) hold a doctorate degree. Concerning the scientific position, (43.1%, 36.1%, and 20.8%) of the participants were assistant professor, lecturer, and associate professor, respectively. Participants’ affiliations were distributed at eight universities as (25%, 18.1%, 16.7%, 15.3%, 8.3%, 8.3%, 5.6%, and 2.8%) at (University of Baghdad, University of Mosul, University of Kufa, University of Kirkuk, University of Al-Muthanna, University of Dhighar, University of Basra, University of Misan), respectively. The mean of participants’ years of experience in academia was 12.23 years with SD of 5.8. The participants’ demographic information is shown in Table 1.

**Table 1:** Participants’ Demographics

	Frequency	Percent	Cumulative Percent
Gender			
Male	37	51.4	51.4
Female	35	48.6	100.0
Marital Status			
Single	10	13.9	13.9
Married	60	83.3	97.2
Divorced	2	2.8	100.0
Academic Degree			
Master’s Degree	36	50.0	50.0
Doctorate Degree	36	50.0	100.0
Scientific Position			
Lecturer	26	36.1	36.1
Assistant Professor	31	43.1	79.2
Associate Professor	15	20.8	100.0
Monthly Income			
Not Enough	4	5.6	5.6
Mostly Enough	21	29.2	34.7
Enough	47	65.3	100.0

Regarding the variables that interface with participants’ health such as smoking, drinking alcohol, obesity, and having chronic disease; the data is informed in Table 2. The data revealed that most of the participants (81.9%) do not smoke. Also, no one (0%) drinks alcohol. About classifications of BMI, (31.9%, 38.9%, and 29.2%) of the participants were (normal weight, overweight, and obese), respectively. Most of the participants (77.8%) reported they do not suffer from any chronic disease while 13.9% and 8.3% reported hypertension and diabetes mellitus, respectively.

**Table 2:** Participants’ Health Status

	Frequency	Percent	Cumulative Percent
Smoking			
No	59	81.9	81.9
Yes	13	18.1	100.0
Drinking Alcohol			
No	72	100	100
Yes	0	0	100.0
BMI			
Normal Weight	23	31.9	31.9
Overweight	28	38.9	70.8
Obese	21	29.2	100.0
Chronic Disease			
None	56	77.8	77.8
Hypertension	10	13.9	91.7
Diabetes	6	8.3	100.0

Concerning HPLP-II, the mean of the overall scale and means of the six domains are shown in Table 3. The mean of the domains *“health responsibility”, “physical activity”, “nutrition”, “spiritual growth”, “interpersonal relations”, and “stress management*” were (2.4954, SD 0.5894; 1.8941, SD 0.63588; 2.6250, SD 0.51863; 3.1250, SD 0.69325; 2.9568, SD 0.56118; 2.3559, SD 0.57518), respectively. The mean of overall HPLP-II was 2.5754 with SD of 0.40458 for the all 52 items. These results indicate that the nursing faculty in this study reported low health promotion in the domains of *“health responsibility*”; *“physical activity”*; and *“stress management*”. Moderate health promotion was observed in two domains: “*nutrition”* and “*interpersonal relations”*. The participants stated high health promotion in *“spiritual growth”*. In overall, the study sample reported moderate level of health promotion based on HPLP-II.

**Table 3:** The overall mean of HPLP-II and its six domains

	N	Mean	Std. Deviation
Health responsibility	72	2.4954	.58940
Physical activity	72	1.8941	.63588
Nutrition	72	2.6250	.51863
Spiritual growth	72	3.1250	.69325
Interpersonal relations	72	2.9568	.56118
Stress management	72	2.3559	.57518
Overall HPLP-II	72	2.5754	.40458

The test of Independent two-sample t test was used to test the difference between independent dichotomous variables of study sample’s demographics with domains of HPLP II instrument while analysis of variance (ANOVA test) was used for polychotomous independent variables of demographic characteristics with domains of HPLP II instrument. A statistically significant difference was found (t = -2.07, p =.040; t = 2.07, p =.041) between domains of nutrition and stress management respectively and gender variable. Furthermore, Interpersonal relations, and Stress management domains of HPLPII significantly differ (F = 4.43, p= .015; F = 3.73, p = .029) with participants’ scientific position. The results also showed that spiritual growth domain significantly differ with regard to years of experience in nursing variable (F = 2.393 p = .019). Moreover, there was a statistically significant difference (t = 3.54, p =.001; t = 2.19, p= .031) between smoking and nutrition and spiritual growth domains of HPLPII respectively. The results of the current study did not reveal statistically significant differences between following demographic variables, which are (age groups marital status, affiliation, academic degree, BMI, and chronic disease history) and domains of HPLP II at p < 0.05 (See Table 4).

**Table 4:** Comparison of HPLP-II Domains with Demographic Characteristics of the Study Sample

Domains Demographics	Health responsibility		Physical activity		Nutrition		Spiritual growth		Interpersonal relations		Stress management	
	Test Value	*p* value	Test Value	*p* value	Test Value	*p* value	Test Value	*p* value	Test Value	*p* value	Test Value	*p* value
Gender	.66	.51	.94	.34	-2.07	**.04**	.92	.36	.81	.42	2.07	**.041**
Age	1.3	.19	1.2	.22	.32	.74	.92	.35	1.17	.24	-.63	.52
Marital status	.47	.62	.14	.86	.41	.66	.15	.85	.47	.62	.63	.53
Affiliation	.75	.624	.87	.53	.58	.76	1.45	.19	1.06	.39	1.9	.07
Academic Degree	-.15	.87	1.28	.2	.27	.78	1.4	.14	1.6	.11	1.2	.21
Scientific position	1.04	.35	.61	.54	.72	.49	2.2	.11	4.4	**.015**	3.7	**.029**
Years of experience	1.8	.07	.51	.61	1.25	.21	2.39	**.019**	1.7	.09	.2	.84
Smoking	.17	.86	1.39	.16	3.54	**.001**	2.19	**.031**	1.09	.27	.79	.42
BMI	.78	.505	1.56	.2	.405	.75	.62	.6	.43	.73	1.7	.16
Chronic disease	1.24	.29	2.77	.06	1.94	.15	.51	.59	.43	.65	1.72	.18

**Table 5:** Comparison of overall HPLPII by demographic characteristics of studied samples

No.	Demographic variable	Test value	*p* value
1.	Gender	0.51	0.603
2.	Age groups	2.06	0.135
3.	Marital status	0.39	0.677
4.	Affiliation	1.38	0.227
5.	scientific Degree	2.02	0.159
6.	Scientific position	3.76	0.028
7.	Years of experience in nursing	4.11	0.046
8.	Smoking	2.13	0.036
9.	BMI	0.44	0.719
10.	Chronic disease history	1.15	0.321

Analysis of variance (ANOVA test) was used to test difference between polychotomous independent variables of demographic characteristics with overall HPLP II instrument, while the test of Independent two-sample t test was used to test the difference between independent dichotomous variables of study sample’s demographics with overall HPLP II score. A statistically significant difference was found between following demographic variables; scientific position, years of experience in nursing, and Smoking and overall HPLP II score at p < 0.05 respectively (F= 3.762 p = 0.028, F= 4.117 p = 0.046, t = 2.139 P = 0.036)

## DISCUSSION

Healthy Lifestyle (HL) is cornerstone of human health, which has well-demonstrated role to reduce “all-cause mortality “and to maintain health and wellbeing of individuals^(12,17)^, so it is highly significant to conduct study addressing health lifestyle behaviors of nursing faculty in Iraq, due to their importance in nursing as profession especially during the pandemic of COVID-19. due to this pandemic has effect on individual lifestyle^(12)^.

The current findings showed approximately two third of studied sample were within obese and overweight classes regarding their BMI, also the current study findings showed that nursing faculty had low physical activity level, it may be inferred according to the these findings that decreased physical activity was predictor of increased BMI. Furthermore, less than one quarter of the current sample of the study had history of chronic diseases in terms of hypertension and diabetes mellitus which make them susceptible more to incidence with cardiovascular diseases specifically CAD ^(18)^. To decrease cardiovascular risk in this specific population with cardiometabolic disorder like diabetes mellitus, it is advisable to follow HL as prevention and management strategy ^(12)^.

Findings from study conducted in South Korea reported that adults during pandemic had low physical activity thereby it is considered negative effect due to COVID 19 pandemic, in turn this may lead to increase of physical diseases most importantly cardiovascular diseases^(11)^. Similarly, The study results stated that nursing faculty had low level of physical activity. This can be attributed to some factors that make them physically inactive. One of them is the online teaching during the pandemic of Covid-19. Nursing faculty used to go to classes; library; or hospitals for clinical training, and these activities require a lot of movement and walking. However, during the pandemic, they teach from home via electronic platforms which make them physically inactive. Other significant factor that decreases physical activity among nursing faculty can be closing the gyms and sport facilities by the government to reduce the spread of corona virus. In addition, most of the Iraqi people who live in cities cannot practice sport activities like cycling or running outdoor due to crowd and unpaved streets. In a consistent result from a study conducted in Jordan for subjects without the history of CHD who reported low physical activity^(2)^.

Concerning the health responsibility domain, the findings of the study revealed a low mean score, which is considered significant predictor for incidence with CVDs. It is surprising that nursing faculty do not have enough attention to their health. The more dangerous point is that a nursing faculty is a symbol for his/her students, family, and friends. Poor health responsibilities can increase the chance of developing CVDs. Eshah (2013) found that patients with acute coronary syndrome reported low health responsibilities^(16)^.

Regarding to stress management domain, the study sample showed a low score in stress management. Facing new changes in life based on the pandemic can lead to stress and manage that stress may not be easy. This result was supported by a cross-sectional study in Kuwait. The study stated the impact of COVID-19 upon lifestyle behaviors of the study sample, and that can affect consequently on cardiovascular health^(13)^.

A cohort study was conducted in china to study the relationship between CVDs and HPLBs. The results found a strong relationship between practicing HPLBs and CVDs. In addition, that study showed the sample had poor adherence to HPLBs and suffered from CVDs^(6)^, Supporting evidence confirm this association that HLs are related to multiple non-communicable diseases (NCDs), cardiovascular diseases as example^(17)^. The current study declared that the study sample had moderate level of HPLBs which put nursing faculty at risk for CVDs, especially coronary heart disease.

The current findings demonstrated that there is statistically significant relationship between scientific position and HPLBs. It may be interpreted that difference in level of scientific position impact level of lifestyle practices especially in nursing profession where nursing teachers have knowledge at uneven levels about prevention and management of diseases as general. Based on previous mentioned outcomes of lifestyle research studies, it is highly advisable for nursing teacher to be familiar and more knowledgeable toward HPLBs.

### Limitations

The descriptive design can be a limitation in this study. Also, the results of the study cannot be generalized to the whole nursing faculty as the response rate was low which made the sample size to be small. The sampling method was purposive sampling which may also affect the generalization of the results. On the other hand, collecting the data online and via Google Form cannot guarantee data protection although collecting the data personally was not possible based on the restrictions during the pandemic.

## CONCLUSION

This is the first informative study about studying healthy lifestyle behaviors of nursing faculty during the pandemic of COVID-19. Nursing faculty stated a moderate level of HPLPs, and this indicates that they can be at risk of developing CVDs. Low physical activity, low health responsibility, and low stress management are domains that were stated by the study sample. These factors can significantly increase the risk of developing CVDs. To reduce the chance of developing CVDs among nursing faculty, enhancing the lifestyle behaviors is crucial. This can be done by encourage nursing faculty to maintain normal body mass index, cease smoking, control stress, and increase physical activity. It is recommended to conduct more studies with large samples to find out the factors that affect lifestyle behaviors in order to prevent the incidence of CVDs.
